# Crystal Structure and Density Functional Theory Study on Structural Properties and Energies of a Isonicotinohydrazide Compound

**DOI:** 10.3390/molecules16097715

**Published:** 2011-09-08

**Authors:** Hajar Sahebalzamani, Nina Khaligh, Shahriar Ghammamy, Farshid Salimi, Kheyrollah Mehrani

**Affiliations:** 1 Department of Chemistry, Faculty of Science, Ardabil Branch, Islamic Azad University, Ardabil, Iran; 2 Department of Chemistry, Facutly of Science, Takestan Branch, Islamic Azad University, Takestan, Iran

**Keywords:** isonicotinohydrazide, DFT, IR, HOMO, LUMO

## Abstract

An X-ray and a theoretical study of the structure of the isoniazid derivative *N'*-(4-dimethylaminobenzylidene)-isonicotinohydrazide monohydrate (**1**) are reported. In this work, we will report a combined experimental and theoretical study on the molecular structure, vibrational spectra and energies of *N'*-(4-dimethylaminobenzylidene)-isonicotinohydrazide monohydrate. The calculated parameters are in good agreement with the corresponding X-ray diffraction values. The FTIR spectrum in the range of 400–4000 cm^−1^ of *N'*-(4-dimethylaminobenzylidene)-isonicotinohydrazide monohydrate has been recorded. The molecular geometry and vibrational frequencies and energies in the ground state are calculated by using the DFT (B3LYP, PBE1PBE) methods with 6-311G** basis sets. The calculated HOMO and LUMO energies also confirm that charge transfer occurs within the molecule. The geometries and normal modes of vibrations obtained from B3LYP/PBE1PBE/6-311G** calculations are in good agreement with the experimentally observed data.

## 1. Introduction

Heterocyclic nitrogen-containing compounds, such as pyridine and its derivatives, are commonly present in synthetic and natural products [1,2]. The study of the vibrational spectra of substituted pyridines, mainly aminopyridines, has attracted the attention of many spectroscopists due to their wide application in pharmacology and agrochemistry. Pyridine heterocycles are a repeated moiety in many large molecules with interesting photophysical, electrochemical and catalytic applications [3,4,5,6,7,8]. They serve as good anesthetic agent and hence are used in the preparation of drugs for certain brain diseases. These pharmaceutically acceptable salts and the pre-drugs are used for the treatment (or) prevention of diabetic neuropathy [9].

The chemistry of Schiff bases has been intensively investigated in recent years, owing their coordination properties and diverse applications. Schiff base hydrazones are widely used in analytical chemistry as selective metal extracting agents as well as in spectroscopic determination of certain transition metals [10,11]. Schiff bases play an important role in inorganic chemistry as they easily form stable complexes with most transition metal ions in the periodic table. The development of the field of bioinorganic chemistry has increased the interest in Schiff base complexes, since it has been recognized that many of these complexes may serve as models for biologically important species. Schiff base metal complexes have been widely studied because they have industrial, fungicide, antibacterial, anticancer and herbicidal applications [12,13].

However, the detailed B3LYP/BPBE1PBE at 6-311G**comparative studies on the complete FTIR spectra of *N'*-(4-dimethylaminobenzylidene)-isonicotinohydrazide monohydrate have not been reported so far. In this study, molecular geometry, optimized parameters and vibrational frequencies, energies are computed and the performance of the computational methods for hybrid density functional methods (B3LYP and BPE1PBE) at 6-311G** basis sets are compared.

## 2. Results and Discussion

### 2.1. Molecular Geometry

**Figure 1:**
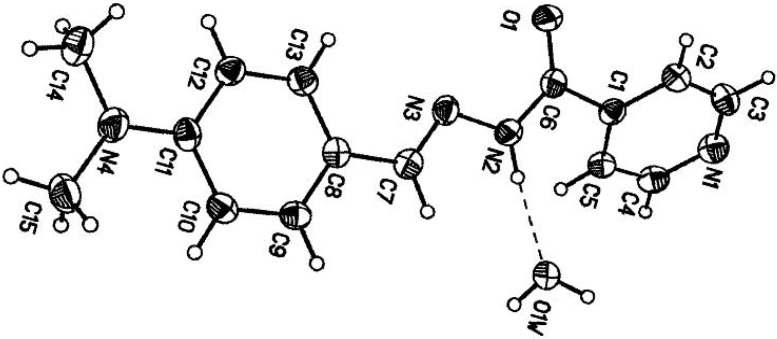
ORTEP diagram of (1) with ellipsoids drawn at the 50% probability level.

The molecular structure and atom numbering of *N'*-(4-dimethylaminobenzylidene)-isonicotinohydrazide monohydrate are shown in Figure 1. The crystal data, details concerning data collection and structure refinement are given in Table 1. The O atom and the hydrazinic N3 atom are *cis* with respect to C6–N2 bond. The structure of the compound reveals the quasi coplanarity of the whole molecular skeleton with localization of the double bonds in the central –C=N–N–C=O which has an *E*-configuration with respect to the double bond of the hydrazone bridge. A *trans s-cis* configuration is fixed around the N3–N2 (1.398 Å) single bond [14]. The angle O1–C6–N2 (125.0) is significantly greater than O1–C6–C1 (119.9) possibly in order to relive repulsion between lone pairs of electrons on atoms N3 and O1. The central part of the molecule C7–N3–N2–C6–C1, adopts a completely extended conformation. The bond lengths C7–N3 (1.284 Å) and C6–O1 (1.242 Å) are typical of double bonds, so that the chain likely corresponds to C7=N3–N2–C6=O1.

**Table 1:** Crystal data and structure refinement for compound **1**.

Identification code	119
Empirical formula	C_15_H_18_N_4_O_2_
Formula weight	286.33
Temperature	120(2) K
Wavelength	0.71073 Å
Crystal system	Orthorhombic
Space group	P 2_1_ 2_1_ 2_1_
Unit cell dimensions	a = 7.2272(17) Å
	b = 11.745(3) Å
	c = 17.177(5) Å
Volume	1458.1(7) Å 3
Z	4
Density (calculated)	1.304 Mg/m3
Absorption coefficient	0.090 mm^−1^
F(000)	608
Crystal size	0.34 × 0.08 × 0.06 mm3
Theta range for data collection	2.10 to 28.00°
Index ranges	−9 ≤ h ≤ 9, −15 ≤ k ≤ 15, −22 ≤ l ≤ 22
Reflections collected	15039
Independent reflections	2034 [R(int) = 0.0529]
Completeness to theta = 28.00°	100.0%
Absorption correction	None
Refinement method	Full-matrix least-squares on F2
Data / restraints / parameters	2034 / 0 / 192
Goodness-of-fit on F2	0.998
Final R indices [for 1694 rfls with I>2sigma(I)]	R1 = 0.0419, wR2 = 0.0848
R indices (all data)	R1 = 0.0562, wR2 = 0.0944
Largest diff. peak and hole	0.200 and −0.181 e. Å −3

In the crystal structure, molecules are linked through intermolecular N–H···O, O–H···N and O–H···O hydrogen bonds involving the water molecule. The crystal structure determination of the title compound was undertaken as part of a study to investigate the physical and chemical properties of the compound. The latter value suggests that intermolecular hydrogen bond (Table 2) stabilizes the planar conformation of part of the molecule.

**Table 2:** Hydrogen bonds for compound (1) [Å and °].

D-H···A	d(D-H)	d(H···A)	d(D···A)	<(DHA)
N(2)-H(2B)···O(1W)#1	0.86	1.898	2.730(3)	162
O(1W)-H(1)···O(1)#2	0.85	1.962	2.717(3)	147
O(1W)-H(2)···N(1)#3	0.85	1.977	2.818(3)	170

**Figure 2:**
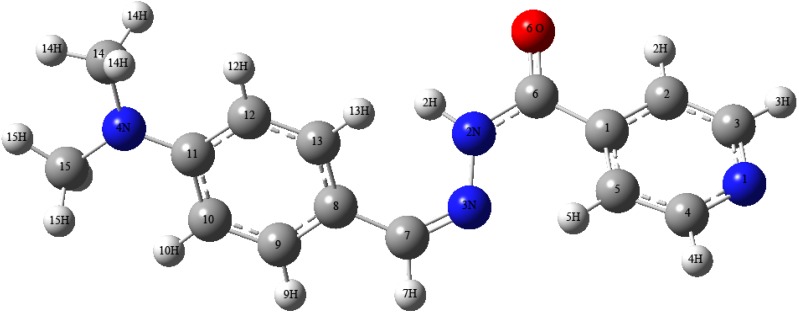
Numbering system adopted in the study for **1** using B3LYP/6-311G**.

The optimized molecular structure of title molecule is obtained from GAUSSAN 03W and GAUSSVIEW programs is shown in Figure 2.

The optimized structural parameters of compound **1** calculated by B3LYP/PBE1PBE levels with the 6-311G** basis set are listed in Table 3 and Table 4. From the theoretical values, it is found that most of the optimized bond lengths are slightly larger than the experimental values, due to that the theoretical calculations belong to isolated molecules in gaseous phase and the experimental results belong to molecules in solid state.

**Table 3:** Comparison of calculated bond length (Å) for the compound **1**.

Bond lengths	X-ray	B3LYP	PBE1PBE
C(1)-C(2)	1.383(3)	1.403	1.399
C(1)-C(5)	1.392(3)	1.402	1.397
C(1)-C(6)	1.504(3)	1.493	1.487
C(3)-N(1)	1.342(3)	1.352	1.347
C(4)-N(1)	1.338(3)	1.350	1.345
C(2)-C(3)	1.382(3)	1.392	1.388
C(4)-C(5)	1.388(3)	1.395	1.392
C(6)-O(1)	1.236(3)	1.255	1.250
C(6)-N(2)	1.342(3)	1.378	1.370
C(7)-N(3)	1.290(3)	1.301	1.297
C(7)-C(8)	1.458(3)	1.467	1.462
C(8)-C(9)	1.398(3)	1.410	1.405
C(8)-C(13)	1.402(3)	1.411	1.406
C(9)-C(10)	1.382(3)	1.386	1.383
C(10)-C(11)	1.410(3)	1.419	1.414
C(11)-N(4)	1.370(3)	1.384	1.376
C(11)-C(12)	1.415(3)	1.418	1.413
C(12)-C(13)	1.385(3)	1.389	1.385
C(14)-N(4)	1.454(3)	1.464	1.452
C(15)-N(4)	1.453(3)	1.464	1.452
N(2)-N(3)	1.398(3)	1.388	1.374

**Table 4:** Comparison of calculated bond angles (°) for the compound (1).

Bond angles	X-ray	B3LYP	PBE1PBE
C(2)-C(1)-C(6)	118.5(2)	116.6	116.2
C(5)-C(1)-C(6)	122.7(2)	125.2	125.5
N(1)-C(3)-C(2)	123.7(2)	122.9	123.0
N(1)-C(4)-C(5)	123.8(2)	123.4	123.5
O(1)-C(6)-N(2)	125.0(2)	117.7	116.8
O(1)-C(6)-C(1)	119.9(2)	120.7	120.6
N(2)-C(6)-C(1)	115.1(2)	121.6	121.6
N(3)-C(7)-C(8)	122.2(2)	131.2	130.3
C(13)-C(8)-C(7)	122.6(2)	124.5	124.0
N(4)-C(11)-C(10)	121.7(2)	121.4	121.3
N(4)-C(11)-C(12)	121.2(2)	121.4	121.3
C(4)-N(1)-C(3)	116.7(2)	117.5	117.5
C(6)-N(2)-N(3)	119.3(2)	124.2	124.4
C(7)-N(3)-N(2)	113.7(2)	119.2	119.0
C(11)-N(4)-C(15)	120.6(2)	120.3	120.1
C(11)-N(4)-C(14)	121.3(2)	120.3	120.1
C(15)-N(4)-C(14)	118.1(2)	119.4	119.7

According to the experimental values for the pyridine ring, the bond lengths N1–C3 and N1–C4 are (1.342, 1.338 Å) whereas in the case of DFT calculation, the value of bond length N1–C3 is 0.002 Å at B3LYP/6-311G** level and 0.002 Å at PBE1PBE/6-311G**, greater than the N1–C4 bond length. This increase of bond length is exactly at the substitution place and also may be due to the single (C–N) and double (C=N) bond lengths in the ring. Bond lengths of all pairs decrease in going from PBE1PBE/6-311G** to B3LYP/6-311G**.

### 2.2. IR Spectrum

**Figure 3:**
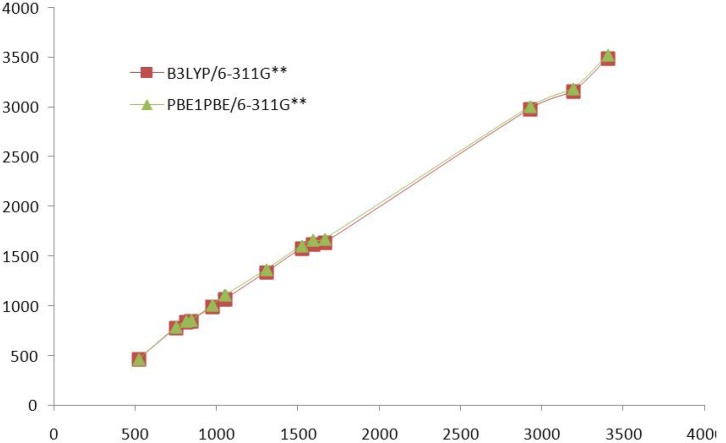
Comparative graph of computed frequencies [DFT] with experimental values for compound **1**.

Vibrational spectroscopy is extensively used in organic chemistry for the identification of functional groups of organic compounds, the study of molecular conformations, reaction kinetics, *etc.* The observed and calculated data of the vibrational spectrum of compound **1** are given in Table 5. The comparative graph of calculated vibrational frequencies by DFT methods at B3LYP/6-311G** and PBE1PBE/6-311G** basis sets for compound **1** are given in the Figure 3.

The suggested reason was that the result obtained by the calculation was harmonic oscillation frequency, while the experimental value contained the anharmonic oscillation frequency. Assignment of compound systems could be proposed on the basis of frequency agreement between the computed harmonics and the observed fundamental modes. The prominent peaks around 3404, 1664 and 1593 cm^−1^ in the FT-IR spectra are attributed to ν_N–H_, ν_C=O_ and ν_C=N_ modes, respectively. The observation of lower ν_C=O_ around 1664 cm^−1^ is due to extended conjugation of C=O group with the nearby pyridine ring. The C=C stretching vibration of the aromatic ring appeared around 1524 cm^−1^. The peaks around 1055 and 974 cm^−1^ are due to ν_N–N_ and aromatic C–H out of plane bending vibrations, respectively. The in plane bending vibration of methyl group are characterized by bands in the range of 1308 cm^−1^, respectively.

**Table 5:** Theoretical and experimental IR spectral data (cm^−1^) of compound (1).

Freq.	Int. (IR)	B3LYP 6-311G**	Int. (IR)	PBE1PBE 6-311G**	Int. (IR)	Vib.
3407	m	3490	30.44	3523	36.83	ν_N–H_
3191	m	3157	18.03	3184	14.14	ν_C–H_ (aromatic)
2928	m	2981	12.33	3002	97.84	ν_C–H_ (CH_3_ symmetric)
1664	s	1633	181.51	1673	216.11	ν_C=O_
1593	s	1617	168.64	1661	200.13	ν_C=N_
1524	s	1577	16.21	1607	16.42	ν_C=C_ (aromatic)
1308	s	1340	148.77	1369	112.49	C–H methyl in plane
1055	m	1068	55.76	1107	34.71	ν_N–N_
974	w	989	1.56	1009	1.44	C–H C–H out of plane
846	w	851	35.56	859	31.32	C–H
813	m	836	16.82	843	22.38	C–H
750	w	775	50.37	785	69.11	C–H
524	w	464	7.80	467	7.94	C–H

### 2.3. Orbital Analysis

**Figure 4:**
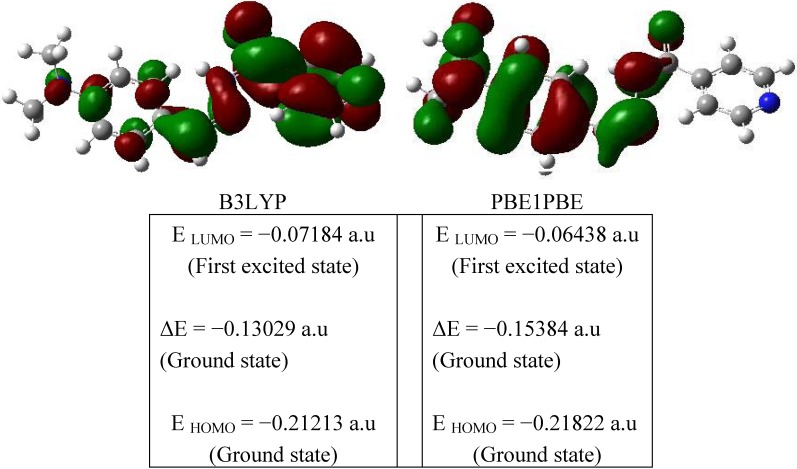
The atomic orbital compositions of the frontier molecular orbital for compound (1).

All the structures allows strong σ → σ* or π → π* transitions in the UV-Vis region with high extinction coefficients. On the basis of fully optimized ground-state structure, B3LYP/PBE1PBE levels with the 6-311G** basis set calculations have been used to determine the low-lying excited states of *N'*-(4-dimethylaminobenzylidene)isonicotinohydrazide monohydrate. Both the highest occupied molecular orbital (HOMO) and lowest unoccupied molecular orbital (LUMO) are the main orbitals that participate in chemical stability [15]. The HOMO represents the ability to donate an electron, LUMO as an electron acceptor represents the ability to obtain an electron. The HOMO and LUMO energy calculated by B3LYP/PBE1PBE levels with the 6-311G** basis set are shown in Figure 4. This electronic absorption corresponds to the transition from the ground to the first excited state and is mainly described by one electron excitation from the highest occupied molecular or orbital (LUMO). The HOMO is located over the group, the HOMO → LUMO transition implies an electron density transfer to ring from chlorine and partially from ring.

## 3. Experimental, Theoretical and Computational Methods

### 3.1. General

All chemicals were purchased from Merck and were used without further purification. Nuclear magnetic resonance spectra were recorded using a Bruker Avance 500 MHz instrument. All the chemical shifts are quoted in ppm using the high-frequency positive convention; ^1^H-NMR spectra were referenced to external TMS. Melting points were determined in an Electrothermal 9200 apparatus. Infrared spectra (400–4000 cm^−1^), were recorded using FT-IR Bruker Tensor 27 spectrophotometer at room temperature. The samples were prepared as KBr pellets.

### 3.2. Preparation of N'-(4-Dimethylaminobenzylidene)isonicotinohydrazide Monohydrate

*N'*-(4-dimethylaminobenzylidene)-isonicotinohydrazide monohydrate (**1**) was synthesized starting from a hot solution of isonicitinic acid hydrazide salt (1.371 g, 1 mmol) in MeOH (100 mL) to which was added a stoichiometric amount (equimolar) of 4-dimethylaminobenzaldehyde (0.149 g, 1 mmol). The mixture was refluxed for about 24 h. After removal of the methanol the yellow solid that appeared was filtered off, and washed three times with dichloromethane and dried in vacuum at 80 °C for 24 h. m.p. 194.7–196.6 °C, ^1^H-NMR (500 MHz, CDCl_3_) δ: 9.7 (1H, s, NH), 7.7 and 7.6 (1H, d, pyridine), 7.2 and 6.7 (1H, d, C_6_H_6_), 8.2 (1H, s, NCH), 2.9 (3H, s, CH_3_); IR ((KBr) *ν*: 3407 (N–H), 2928 (C–H), 1664 (C=O), 1593 (C=N), 1524 (C=C), 1055 (N–N). Anal. Calcd. for C_15_H_16_N_4_O: C 67.1, H 5.9, N 20.8, found: C 68.1, H 6.1, N 21.2.

### 3.3. X-ray Crystallography

A yellow crystal with approximate dimensions of 0.34 × 0.08 × 0.06 mm^3^ was selected for data collection. The X-ray diffraction data were collected on a Bruker SMART APEX II CCD diffractometer equipped with a graphite monochromated Mo Ka radiation (k = 0.71073 Å) by using phi and omega scans technique at room temperature. The structure was solved by direct methods with SHELXS-97 [16], and refined using the full-matrix least squares method on F^2^ with anisotropic thermal parameters for all non-hydrogen atoms using SHELXL-97 [17]. Hydrogen atoms were generated geometrically. Positions of H(O) and H(N) atoms were found in difference Fourier maps and of H(C) were calculated. All hydrogen atoms were refined in isotropic approximation in riding model with the Uiso(H) parameters equal to 1.5 Ueq(Ci) for methyl groups, 1.5 Ueq(Oi) for water molecule and to 1.2 Ueq(Xi) for other atoms, were Ueq(X) are the equivalent thermal parameters of the atoms to which the corresponding H atoms are bonded. Parameters in CIF format are available as Electronic Supplementary Publication from the Cambridge Crystallographic Data Centre (CCDC 698644).

### 3.4. Computational Methods

All calculations were performed using the Gaussian 03 package of program [18] on a Windows-XP operating PC. The molecular structure of the title compound in the ground state is computed by performing both DFT (B3LYP/PBE1PBE) with 6-31G** basis sets. Full optimization for the all molecules were carried out by the DFT method using B3LYP—The dynamical functional of Lee, Yang, and Parr (LYP), coupled with Becke’s three-parameter pure DFT exchange functional (B3) [19], and PBE1PBE hybrid functional of Perdew, Burke and Ernzerhof which uses 25% exchange and 75% correlation weighting, with the 6-311G** basis set.

## 4. Conclusions

The structure of *N'*-(4-dimethylaminobenzylidene) isonicotinohydrazide monohydrate has been determined by single-crystal X-ray diffraction and its geometry was compared with optimized parameters obtained by means of Density Functional Theory calculations at the B3LYP/PBE1PBE levels with the 6-311G** basis set. A good agreement between theory and X-ray diffraction was found.

## Supplementary Materials

Supplementary materials can be accessed at http://www.mdpi.com/1420-3049/16/9/7715/s1.

## Supplementary Materials

Supplementary File 1
