# Supplementary material for: Crystal Structure and Density Functional Theory Study on Structural Properties and Energies of a Isonicotinohydrazide Compound
Source: Molecules. 2011 Sep 8;16(9):7715–24. doi: 10.3390/molecules16097715 (PMC6264359; doi:10.3390/molecules16097715)
Supplement: Supplementary File 1 [file molecules-16-07715-s001.pdf]

Supplementary Materials

Table S1. XYZ coordinates for compound (1) by using DFT/B3LYP/6−311G**.

Center Number	Number Atomic	Type Atomic	Coordinates (Angstroms) X	Y	Z
1	6	0	−3.008572	0.761847	0.599093
2	6	0	−1.712612	0.262390	0.606260
3	6	0	−1.411727	−1.024142	0.108721
4	6	0	−2.494770	−1.796217	−0.361191
5	6	0	−3.792700	−1.307995	−0.385689
6	6	0	−4.088147	−0.001326	0.084215
7	1	0	−3.182527	1.746189	1.005316
8	1	0	−0.938247	0.869115	1.056349
9	1	0	−2.307858	−2.800681	−0.722219
10	1	0	−4.579127	−1.941639	−0.765586
11	7	0	−5.377046	0.502187	0.054148
12	6	0	−6.476562	−0.313768	−0.464417
13	1	0	−7.396857	0.262481	−0.422723
14	1	0	−6.306954	−0.603507	−1.505979
15	1	0	−6.620748	−1.224567	0.125822
16	6	0	−5.656015	1.848315	0.558785
17	1	0	−6.712225	2.065579	0.425083
18	1	0	−5.424060	1.939762	1.624880
19	1	0	−5.084125	2.607600	0.017005
20	6	0	−0.075639	−1.629571	0.118765
21	1	0	−0.038047	−2.706064	0.246074
22	7	0	1.106509	−1.104472	−0.031557
23	7	0	1.216264	0.248856	−0.309939
24	1	0	0.393167	0.781921	−0.575129
25	6	0	2.390981	0.961486	−0.431508
26	8	0	2.284157	2.163074	−0.787556
27	6	0	3.739409	0.379836	−0.133426
28	6	0	4.817108	1.281729	−0.175046
29	6	0	4.021688	−0.962042	0.166750
30	6	0	6.104833	0.822950	0.082978
31	1	0	4.627637	2.318260	−0.409934
32	6	0	5.346457	−1.334044	0.407703
33	1	0	3.229807	−1.688980	0.209630
34	7	0	6.382717	−0.469723	0.373134
35	1	0	6.950423	1.495957	0.060406
36	1	0	5.594979	−2.360649	0.638977

Table S2. XYZ coordinates for compound (1) by using DFT/PBE1PBE/6−311G**.

Center Number	Number Atomic	Type Atomic	Coordinates (Angstroms)		
			X	Y	Z
1	6	0	−2.948640	0.674252	0.703032
2	6	0	−1.669473	0.142248	0.696670
3	6	0	−1.386235	−1.092111	0.077144
4	6	0	−2.465537	−1.782325	−0.501695
5	6	0	−3.746183	−1.256580	−0.513297
6	6	0	−4.022611	−0.002354	0.080070
7	1	0	−3.115500	1.617259	1.204772
8	1	0	−0.886859	0.675100	1.224241
9	1	0	−2.291159	−2.748670	−0.960924
10	1	0	−4.537889	−1.824115	−0.982016
11	7	0	−5.285961	0.531029	0.063079
12	6	0	−6.370077	−0.187764	−0.578433
13	1	0	−7.281877	0.401400	−0.508349
14	1	0	−6.168401	−0.365627	−1.640814
15	1	0	−6.559807	−1.155957	−0.100826
16	6	0	−5.542922	1.812392	0.692552
17	1	0	−6.590398	2.073973	0.558835
18	1	0	−5.338822	1.788135	1.769291
19	1	0	−4.939256	2.612105	0.249307
20	6	0	−0.050754	−1.700558	0.075432
21	1	0	0.006646	−2.781184	0.154001
22	7	0	1.109584	−1.135310	−0.041374
23	7	0	1.159400	0.218988	−0.248078
24	1	0	0.308007	0.727573	−0.476140
25	6	0	2.310587	0.965739	−0.379198
26	8	0	2.194908	2.160681	−0.715417
27	6	0	3.662513	0.397163	−0.118883
28	6	0	4.740071	1.243949	−0.400772
29	6	0	3.945356	−0.873891	0.398363
30	6	0	5.531128	0.798890	−0.165897
31	1	0	4.541197	2.232254	−0.793224
32	6	0	5.279149	−1.231498	0.606948
33	1	0	3.149742	−1.564057	0.616463
34	7	0	6.315622	−0.421203	0.333013
35	1	0	6.888690	−2.203741	1.007147
36	1	0	6.037220	1.430263	−0.377125
